# Multiple-dose up-titration study to evaluate the pharmacokinetics, safety and antitumor activity of apatinib in advanced gastric adenocarcinoma

**DOI:** 10.3389/fonc.2022.876899

**Published:** 2022-10-18

**Authors:** Yonggang Wang, Chang Wang, Yanqiao Zhang, Jiqing Hao, Nong Yang, Jvfeng Wang, Min Peng, Tianshu Liu, Guifang Zhang, Xianbao Zhan, Shan Zeng, Yifan Zhang, Yong Gao, Yang Yao

**Affiliations:** ^1^ Department of Oncology, Shanghai JiaoTong University Affiliated Sixth People’s Hospital, Shanghai, China; ^2^ Tumor Center, The First Hospital of Jilin University, Changchun, China; ^3^ Department of Gastrointestinal Medical Oncology, Harbin Medical University Cancer Hospital, Harbin, China; ^4^ Department of Oncology, The First Affiliated Hospital of Anhui Medical University, Hefei, China; ^5^ Department of Medical Oncology, Lung Cancer and Gastrointestinal Unit, Hunan Cancer Hospital, The Affiliated Cancer Hospital of Xiangya School of Medicine, Changsha, China; ^6^ Department of Internal Medicine, The Affiliated Cancer Hospital of Zhengzhou University, Henan Cancer Hospital, Zhengzhou, China; ^7^ Department of Oncology, Renmin Hospital of Wuhan University, Wuhan, China; ^8^ Department of Oncology, Zhongshan Hospital Fudan University, Shanghai, China; ^9^ Department of Oncology, Xinxiang Central Hospital, Xinxiang, China; ^10^ Department of Oncology, Changhai Hospital, Second Military Medical University, Shanghai, China; ^11^ Department of Oncology, Xiangya Hospital Central South University, Changsha, China; ^12^ State Key Laboratory of Drug Research, Shanghai Institute of Materia Medica, Chinese Academy of Sciences, Shanghai, China; ^13^ Department of Oncology, Shanghai East Hospital, Tongji University School of Medicine, Shanghai, China

**Keywords:** apatinib, angiogenesis, gastric adenocarcinoma, pharmacokinetics, VEGFR, VEGFR-2

## Abstract

**Background and purpose:**

The objective of this study was to investigate the pharmacokinetics, safety, and antitumor activity of apatinib, a vascular endothelial growth factor receptor 2 inhibitor, in advanced gastric adenocarcinoma or gastroesophageal junction adenocarcinoma and evaluate the effect of dose titration on dosage optimization for individual patients.

**Methods:**

Patient with advanced gastric adenocarcinoma progressed after at least one line of chemotherapy were enrolled. Apatinib was given orally once daily starting at 500 mg for 14 days, then up-titrated to 750 mg for 14 days, and then proceeded to a maximum dose of 850 mg. Dose up-titration determination was based on toxicity. The 28-day treatment cycles continued until disease progression, intolerable toxicities, withdrawal of consent, or investigator’ decision.

**Results:**

A total of 60 patients were enrolled, with 17, 18, and 25 patients receiving a maximum dose of 500 mg, 750 mg, and 850 mg, respectively. The pharmacokinetic parameters varied considerably, with the interpatient coefficient of variation for steady state areas under the plasma concentration time curve (AUC_ss_) and the mean maximum concentration of both > 50%. During 500 mg and 750 mg dosing stage, drug exposures in patients with a maximum dosage of 850 mg were lower than in those not titrated to 850 mg. Patients with total gastrectomy exhibited significantly lower AUC_ss_ than patients with partial or no gastrectomy (*p* = 0.004 and 0.032, respectively). Toxicities were tolerable, and disease control rate was 39.5% (95% CI 25.0%−55.6%).

**Conclusions:**

Apatinib dose titration based on toxicity could be used in clinical practice to provide optimal dosage for individual patients.

**Clinical Trial registration:**

https://clinicaltrials.gov/ct2/show/NCT02764268?term=NCT02764268&draw=2&rank=1, NCT02764268.

## Introduction

Apatinib is a small-molecule tyrosine kinase inhibitor (TKI) that highly selectively binds to vascular endothelial growth factor receptor 2 (VEGFR-2), inhibiting tumor angiogenesis (1). Apatinib significantly improved the overall survival (median 6.5 *vs* 4.7 months; hazard ratio [HR] 0.709; *p* = 0.0149) and progression-free survival (PFS; median 2.6 *vs* 1.8 months; HR 0.444; *p* < 0.001) compared with placebo in patients with advanced gastric adenocarcinoma or gastroesophageal junction adenocarcinoma refractory to two or more lines of prior chemotherapy (2). Based on these findings, apatinib was approved in October 2014 by the China National Medical Products Administration for the treatment of this patient population. At the recommended dose of 850 mg once daily, apatinib showed a favorable safety profile, and the adverse events were considered moderate and acceptable compared with other antiangiogenic agents (2–5).

After apatinib came to the market, several studies were conducted and further confirmed the clinical efficacy and safety of apatinib in heavily pretreated patients with gastric cancer (6, 7). Inevitably, as the patient population grows, the heterogeneity in physical condition, renal and hepatic function brings new challenges. In a phase 2 trial (8), high incidence of grade 3 to 4 adverse events was observed in patients with metastatic gastric cancer progressed after two lines of chemotherapy who were given 850 mg of apatinib once daily. Dose reduction occurred in nine of 42 patients due to grade 3 or 4 adverse events and treatment discontinuation occurred in eight patients. The high incidence of adverse events was possibly related with the poor condition of patients enrolled. Additionally, the dose of 500 mg once daily was commonly used in clinical practice due to the concern of grade 3 or 4 adverse events (9). Moreover, a lower dosage of apatinib showed clinical activity and tolerable safety profile in other solid tumors, such as breast cancer, lung cancer, and hepatocellular carcinoma (10–12). Therefore, it is crucial to develop an approach for dose individualization to improve the clinical outcomes of apatinib in patients with advanced gastric cancer.

The previous phase 1 study analyzed the pharmacokinetic (PK) profile of apatinib in patients with advanced solid malignancies (13). However, multiple dosing evaluation was only conducted at 750 mg dose level, and the steady-state PK profiles of 500 mg and 850 mg dose were not adequately described in this trial. In addition, the PK evaluations were derived from a patient population with solid tumors. Therefore, the PK profile of apatinib in gastric cancer remains to be further investigated. We thus conducted this dose up-titration study to investigate the PK profile of apatinib in patients with advanced gastric cancer at doses of 500 mg, 750 mg, and 850 mg once daily, as well as the potential use of dose titration for dose optimization in clinical practice.

## Methods

### Patients

Patients aged between 18 to 70 years, with histologically confirmed advanced gastric adenocarcinoma (including gastroesophageal junction adenocarcinoma) were enrolled. Eligible patients had disease progression after at least two lines of chemotherapy or reluctant to undergo chemotherapy after failure of first-line chemotherapy. Other main eligible criteria included an Eastern Cooperative Oncology Group (ECOG) performance status of 0 or 1; a life expectancy of at least 3 months and adequate organ function. Patients were excluded if they had dysphagia, chronic diarrhea, or intestinal obstruction that might affect swallowing and digestion of oral drugs; had central nervous system metastases; had poorly controlled hypertension; had hemorrhage tendency; participated in other clinical study within four weeks before the first dose; or had received other VEGFR inhibitors.

### Study design, procedures, and objectives

This was a single-arm, open-label, dose up-titration study undertaken in 12 centers in China (ClinicalTrials.gov identifier NCT02764268). Eligible patients were given apatinib orally once daily at a starting dose of 500 mg for 14 consecutive days. The dose was then up-titrated to 750 mg for 14 days, and then proceeded to the maximum dose of 850 mg and maintained at this dose level. If grade ≥ 2 hemorrhage or thromboembolism, or grade ≥ 3 adverse events (except medically controlled hypertension, nausea, or vomiting) occurred during treatment, dose up-titration was halted and the dose level was maintained. Treatment cycles (28 days) were repeated until disease progression, intolerable toxicities, withdrawal of consent, or investigator’ decision. Dose interruption was permitted for grade ≥ 3 adverse events. Patients whose adverse events could not be well managed by dose interruption might have their dose reduced (stepwise to 750 mg, 500 mg, and 250 mg).

The primary objective was to assess PK profile of apatinib at doses of 500 mg, 750 mg, and 850 mg once daily. The secondary objectives were safety and efficacy analyses.

The study was done in accordance with the Declaration of Helsinki and the Good Clinical Practice guidelines. The protocol was approved by the ethics committee at each site. All patients provided written informed consent.

### Assessments

PK samples (2−3 mL) were collected 10 min pre-dose, and 1, 2, 3, 4, 6, 8, 24 h post-dose on days 1 and 14 for the initial 500 mg dose level; then on day 14 for the 750 mg dose level and on day 7 for the 850 mg dose level. For the 850 mg dose level, plasma sampling was stopped after sample collection completed in 20 patients. Under circumstances of treatment interruption, plasma samplings on days 7 or 14 were postponed until steady-state plasma concentration was achieved (after 7 consecutive days of drug administration) (13). Plasma concentrations were determined using liquid chromatograph-mass spectrometer/mass spectrometer method. PK parameters including the maximum plasma concentration (C_max_), time to C_max_ (t_max_), area under the plasma concentration time curve from 0 to 24 h (AUC_24_), plasma concentration at steady state, as well as area under the plasma concentration time curve at steady state (AUC_ss_) were calculated.

Adverse events were graded according to the National Cancer Institute Common Terminology Criteria for Adverse Events (NCI-CTCAE) version 4.03. Efficacy analyses included objective response rate (ORR; defined as the percentage of patients with confirmed complete response or partial response), disease control rate (DCR; defined as percentage of patients with objective response or stable disease lasting at least 4 weeks), PFS (defined as time from first dose to disease progression or death of any cause). Tumor response was assessed every two cycles during treatment. Complete response, partial response, or stable disease need to be confirmed 4 weeks later. Patients were followed up throughout the treatment and 28 days after the last dose of apatinib.

### Statistical analysis

To ensure that at least 20 patients completed sampling at 850 mg dose level, approximately 60 patients were required. If fewer than 20 patients completed sampling at the 850 mg dose level, enrollment continued until 20 patients were up-titrated to the 850 mg dose level and sampled. PK analyses were done in patients who completed all time points PK sampling on any specific sampling day (500 mg day 1, 500 mg day 14, 750 mg day 14, or 850 mg day 7). All patients who received at least one dose of apatinib and had post treatment safety evaluation were included in the safety set. All patients who received apatinib for at least 2 weeks and had at least one tumor response evaluation were included in the efficacy set. PK parameters were descriptively summarized by time and dose level with non-compartmental analysis using WinNonlin 7.0 (Certara, Princeton, New Jersey, USA). Considering gender and gastrectomy history might have an effect on apatinib exposure, subgroup PK parameters were assessed in terms of gender (male *vs* female) and extent of gastrectomy (total gastrectomy *vs* partial gastrectomy *vs* no gastrectomy). An analysis of variance model was used to detect differences in PK parameters among subgroups, and the pair comparisons for C_max_ and AUC in patients with different degrees of gastrectomy were calculated with least significant difference test. Geometric mean ratio of male to female and its 90% confidence intervals (CIs) were given as well. C_max_ and AUC were log_e_ transformed before analysis. For antitumor assessments, 95% CIs for ORR and DCR were estimated using Clopper-Pearson method. PFS was estimated with Kaplan-Meier method. All statistics were performed using SAS version 9.4 (SAS Institute Inc., USA).

## Results

### Patient characteristics

Between January 6, 2017, to October 29, 2018, 60 patients were enrolled. The patient demographics and baseline characteristics are summarized in **Table 1**. The percentage of patients with an ECOG performance status of 1 was 83.3% at baseline, 85.0% of patients were diagnosed with clinical stage IV disease, 98.3% had metastases, 78.3% received primary surgery, and 65.0% experienced at least two lines of chemotherapy. At data cutoff on December 10, 2018, 21 patients (35.0%) discontinued study treatment because of progressive disease (20 patients due to radiographic progression and one patient due to clinical progression). Patient disposition is shown in **Supplementary Table 1**.

**Table 1:** Baseline and disease characteristics.

	Apatinib
	(n = 60)
Age (year), mean (SD)	56 (8.7)
Sex, n (%)	
Female	16 (26.7)
Male	44 (73.3)
Histopathology, n (%)	
Gastric adenocarcinoma	58 (96.7)
Gastroesophageal junction adenocarcinoma	2 (3.3)
ECOG performance status, n (%)	
0	10 (16.7)
1	50 (83.3)
Time since first diagnosis (months), mean (SD)	19.7 (18.5)
Pathological stage, n (%)	
II	12 (20.0)
III	40 (66.7)
Unknown	8 (13.3)
Clinical stage, n (%)	
II	1 (1.7)
III	8 (13.3)
IV	51 (85.0)
Metastases, n (%)	
Yes	59 (98.3)
No	1 (1.7)
No. of metastases organs, n (%)	
1	21 (35.0)
2	24 (40.0)
≥3	14 (23.3)
Previous treatment, n (%)	
Primary surgery	47 (78.3)
Radiotherapy	9 (15.0)
Chemotherapy	60 (100)
Neo-adjuvant	4 (6.7)
Adjuvant	16 (26.7)
First-line	60 (100)
Second-line	36 (60.0)
Third-line	3 (5.0)
Others	8 (13.3)

SD, standard deviation; ECOG, Eastern Cooperative Oncology Group.

### Pharmacokinetics

Plasma concentrations were evaluable in 57 patients at 500 mg day 1, 49 patients at 500 mg day 14, 36 patients at 750 mg day 14, and 22 patients at 850 mg day 7. Thus, 57 patients were included in the PK analysis set. One of the 22 patients up-titrated to 850 mg lacked PK data at 750 mg day 14, and 21 patients completed plasma sampling across all dose ranges from 500 mg to 850 mg.

**Figure 1:**
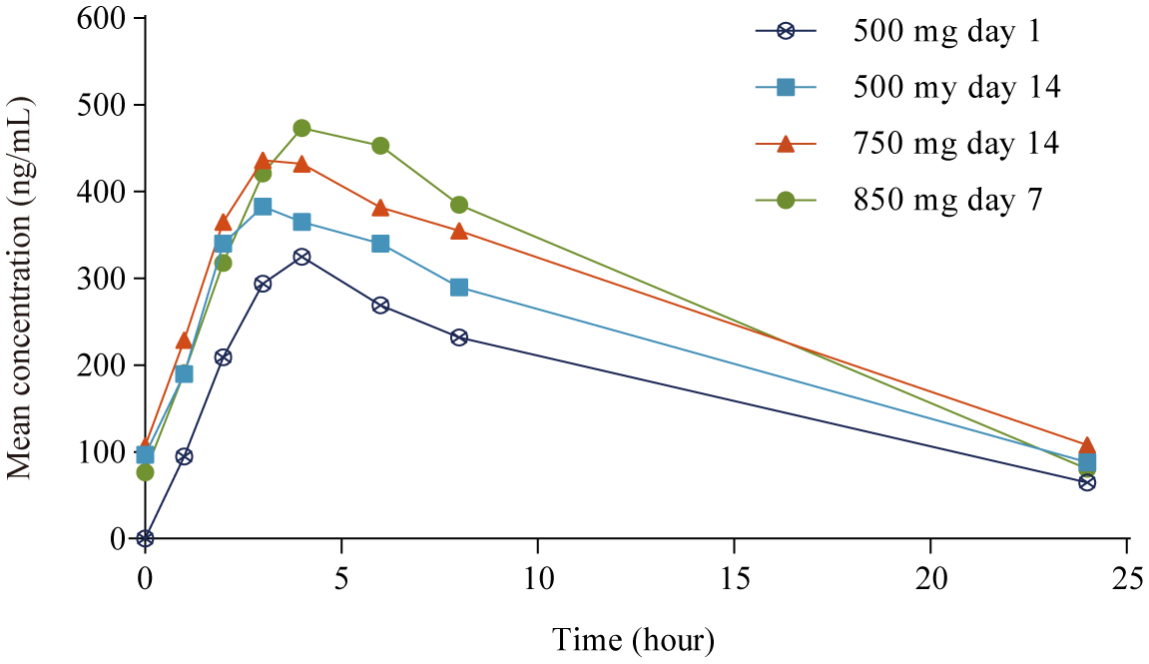
Mean plasma concentration-time profile in pharmacokinetic analysis set.

PK parameters are summarized in **Table 2** and mean plasma concentrations by dose are shown in **Figure 1**. After single dosing (500 mg day 1), the mean C_max_ ± standard deviation (SD) was 386 ± 219 ng/mL. Apatinib exhibited rapid absorption, with the C_max_ observed approximately 4.0 h (range 1.0−8.0 h) after administration. After multiple dosing (500 mg day 14), the mean accumulation ratio (R_ac_) for C_max_ and AUC were 1.56 and 1.61, respectively, suggesting mild drug accumulation with repeated administration. The PK profile varied considerably among patients both after single (500 mg day 1) and multiple dosing (500 mg day 14), with the interpatient percentage of coefficient of variation (CV%) for C_max_ and AUC of 57% and 60% (single dosing), and 59% and 64% (multiple dosing), respectively. In the 21 patients who completed all dose plasma sampling, the mean C_max_ and AUC_ss_ tended to increase as the dose level increased over the 500 mg to 850 mg dose range, whereas increased less proportionally than dose increase (1.0-, 1.5- and 1.7-fold increase in dose, 1.0-, 1.1- and 1.5-fold accumulation in C_max_, and 1.0-, 1.3- and 1.6-fold accumulation in AUC_ss_; **Table 2**). The mean AUC_ss_ ± SD in the 21 patients who completed all dose level plasma sampling was lower than that in the 57 PK evaluable patients at 500 mg day 1 (2980 ± 1830 h·ng/mL *vs* 4210 ± 2510 h·ng/mL), the mean AUC_ss_ ± SD in the 21 patients who completed all dose level plasma sampling was lower than that in the 49 evaluable patients at 500 mg day 14 (4270 ± 2550 h·ng/mL *vs* 5480 ± 3500 h·ng/mL), and the mean AUC_ss_ ± SD in the 21 patients who completed all dose level plasma sampling was lower than that in the 36 evaluable patients at 750 mg day 14 (5420 ± 3320 h·ng/mL *vs* 6560 ± 3910 h·ng/mL).

**Table 2:** Pharmacokinetic parameters of apatinib in patient population assessable at each sampling day and in the 21 patients completed plasma sampling over all dose range.

Dose	Patients (n)	C_max_ (ng/mL)^a^	t_max_ (h)^b^	AUC (h·ng/mL)^c^	R_ac (AUC)_ ^a^	R_ac (Cmax)_ ^a^
500 mg day 1	57	386 ± 219	4.0 (1.0−8.0)	4210 ± 2510	—	—
	21	300 ± 174	4.0 (1.0−8.0)	2980 ± 1830	—	—
500 mg day 14	49	487 ± 288	3.0 (1.0−8.0)	5480 ± 3500	1.61 ± 0.93	1.56 ± 1.13
	21	405 ± 263	4.0 (1.0−8.0)	4270 ± 2550	1.67 ± 0.97	1.70 ± 1.38
750 mg day 14	36	532 ± 258	3.0 (1.0−8.0)	6560 ± 3910	—	—
	21	452 ± 217	3.0 (2.0−8.0)	5420 ± 3320	—	—
850 mg day 7	22	575 ± 332	4.0 (1.0−8.0)	6690 ± 3990	—	—
	21	592 ± 330	4.0 (1.0−8.0)	6850 ± 4020	—	—

C_max_, maximum concentration; t_max_, time to maximum concentration; AUC, area under the plasma concentration-time curve; R_ac(AUC)_, accumulation ratio of AUC; R_ac(Cmax)_, accumulation ratio of C_max_.

a Values are shown as mean ± standard deviation.

b Values are shown as median (range).

c For 500 mg day 1 evaluation, AUC represented AUC_24_; for other evaluations, AUC represented AUC_ss_.

**Figure 2:**
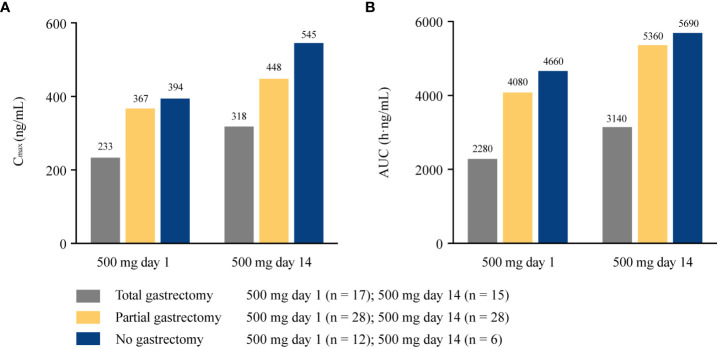
Pharmacokinetic parameters of apatinib in patients with different extent of gastrectomy at 500 mg day 1 and 500 mg day 14. **(A)** C_max_ at 500 mg day 1 and 500 mg day 14; **(B)** AUC_24_ at 500 mg day 1 and AUC_ss_ at 500 mg day 14. C_max_, maximum plasma concentration; AUC_24_, area under the plasma concentration-time curve from time 0 to 24 h; AUC_ss_, areas under the plasma concentration-time curve at steady state.

**Figure 3:**
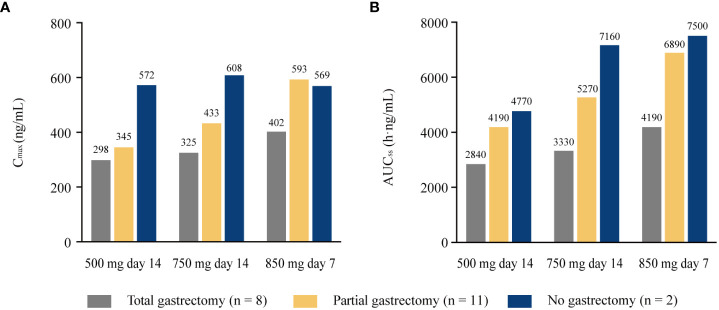
Pharmacokinetic parameters of apatinib in the 21 patients completed all plasma sampling over all dose range with different extent of gastrectomy. **(A)** C_max_; **(B)** AUC_ss_. C_max_, maximum plasma concentration; AUC_ss_, areas under the plasma concentration-time curve at steady state.

The extent of gastric resection significantly influenced apatinib exposure (C_max_ and AUC_24_) after a single dosing of apatinib at 500 mg day 1, with *p* values of 0.026 and 0.001 for C_max_ and AUC_24_ among patients who underwent total gastrectomy (n = 17), partial gastrectomy (n = 28), and no gastrectomy (n = 12), respectively (**Figure 2**). After single dosing (500 mg day 1), C_max_ and AUC_24_ were significantly lower in patients with total gastrectomy than patients with partial gastrectomy (*p* = 0.016 and 0.001 for C_max_ and AUC_24_, respectively; **Supplementary Table 2**). The same trend was observed between patients with total gastrectomy and patients with no gastrectomy (*p* = 0.022 and 0.001 for C_max_ and AUC_24_, respectively). However, there was no significant difference in C_max_ and AUC_24_ between patients with partial gastrectomy and those with no gastrectomy (*p* = 0.726 and 0.491, respectively). At steady-state (500 mg day 14), AUC_ss_ was significantly different among patients with different extent of gastric resection (*p* = 0.010), but C_max_ was similar among subgroups (*p* = 0.079; **Figure 2**). Patients with total gastrectomy showed significantly lower AUC_ss_ compared with patients with partial gastrectomy and no gastrectomy (*p* = 0.004 and 0.032, respectively; **Supplementary Table 3**). However, similar data for AUC_ss_ were observed between patients with partial gastrectomy and with no gastrectomy (*p* = 0.816). Of the 21 patients who completed plasma sampling over dose titration, eight patients underwent total gastrectomy, 11 patients had partial gastrectomy, and two patients had no gastrectomy. C_max_ and AUC_ss_ increased with the increasing dose among all subgroups of patients with total, partial, or no gastrectomy (except for the similar C_max_ among patients without gastrectomy at 750 mg day 14 and 850 mg day 7). The extent of gastrectomy did not obviously influence the percentage increase in apatinib exposure with the increasing doses (**Figure 3**).

There was no statistically significant difference between genders in apatinib exposure (C_max_ and AUC; **Table 3**). After single dosing at 500 mg day 1, geometric mean for C_max_ and AUC_24_ were similar between male and female patients. The geometric mean ratios (male *vs* female) for C_max_ and AUC_24_ were 0.99 (90% CI 0.72−1.36) and 1.10 (90% CI 0.81−1.51), respectively. The steady-state (500 mg day 14) apatinib exposure showed similar results, with the geometric mean ratios for C_max_ and AUC_ss_ of 0.90 (90% CI 0.65−1.23) and 0.92 (90% CI 0.66−1.27), respectively. Similarly, in subgroups of patients with total, partial and no gastrectomy, apatinib exposure (C_max_ and AUC) was not significantly affected by gender differences (**Supplementary Table 4**).

**Table 3:** Pharmacokinetic parameters of apatinib in male and female patients at 500 mg day 1 and day 14.

Dose	Parameters	Geometric mean		Geometric mean ratio (90% CI)	*p* value
		Female	Male	(Male/Female)	
500 mg day 1	AUC_24_ (h·ng/mL)	3283	3621	1.10 (0.81−1.51)	0.6
	C_max_ (ng/mL)	328	324	0.99 (0.72−1.36)	0.96
500 mg day 14	AUC_ss_ (h·ng/mL)	4885	4484	0.92 (0.66−1.27)	0.66
	C_max_ (ng/mL)	448	401	0.90 (0.65−1.23)	0.56

AUC_24_, area under the plasma concentration-time curve from time 0 to 24 h; AUC_ss_, areas under the plasma concentration-time curve at steady state; C_max_, maximum plasma concentration.

### Safety

All 60 patients were included in the safety analysis set. Seventeen patients received a maximum dose of 500 mg (500 mg cohort), 18 patients received a maximum dose of 750 mg (750 mg cohort), and 25 patients received the maximum dose of 850 mg (850 mg cohort). Dose up-titration was stopped for intolerable toxicity in 13 patients each in 500 mg and 750 mg cohorts (**Supplementary Table 5**). The mean dose received ± SD was 469 ± 60 mg in the 500 mg cohort, 575 ± 78 mg in the 750 mg cohort, and 720 ± 80 mg in the 850 mg cohort (**Supplementary Table 6**). The most commonly reported adverse events leading to dose up-titration halt were decreased neutrophil count (5.0%), hand-foot syndrome (5.0%), and asthenia (5.0%; **Supplementary Table 7**). Grade≥3 adverse events occurred in 17 patients (100%) in the 500 mg cohort, 16 patients (88.9%) in the 750 mg cohort, and 16 patients (64.0%) in the 850 mg cohort. Serious adverse events occurred in 12 (70.6%), eight (44.4%), and 10 patients (40.0%), respectively. Dose delay or reduction because of adverse events were reported in 11 patients (64.7%) in the 500 mg cohort, 14 patients (77.8%) in the 750 mg cohort, and 12 patients (48.0%) in the 850 mg cohort. Dose discontinuation or withdrawal due to adverse events were reported in 11 (64.7%), six (33.3%), and four patients (16.0%), respectively.

Grade ≥ 3 adverse events were reported in 49 (81.7%) of 60 patients. The most common grade ≥ 3 adverse events were gamma-glutamyltransferase increased (13.3%), hypertension (11.7%), hyponatremia (8.3%), hand-foot syndrome (8.3%), decreased neutrophil count (8.3%), and increased aspartate aminotransferase (8.3%). Adverse events occurring in at least 10% of patients are listed in **Table 4**. The most commonly reported adverse events of any grade were decreased white blood cell count (48.3%), proteinuria (41.7%), and decreased platelet count (40.0%). Dose interruption or modification because of adverse events was observed in 37 (61.6%) patients. Adverse events leading to dose discontinuation were reported in 15 (25.0%) patients (**Supplementary Table 8**).

**Table 4:** Adverse events occurring in at least 10% of patients.

	All patients (n = 60)	

	Any grade	Grade ≥3
White blood cell count decreased	29 (48.3)	2 (3.3)
Proteinuria	25 (41.7)	3 (5.0)
Platelet count decreased	24 (40.0)	2 (3.3)
Neutrophil count decreased	23 (38.3)	5 (8.3)
Anaemia	23 (38.3)	1 (1.7)
Aspartate aminotransferase increased	22 (36.7)	5 (8.3)
Asthenia	22 (36.7)	4 (6.7)
Hypertension	21 (35.0)	7 (11.7)
Blood bilirubin increased	17 (28.3)	3 (5.0)
Alanine aminotransferase increased	16 (26.7)	3 (5.0)
Hand-foot syndrome	14 (23.3)	5 (8.3)
Diarrhoea	14 (23.3)	1 (1.7)
Gamma-glutamyltransferase increased	13 (21.7)	8 (13.3)
Blood alkaline phosphatase increased	12 (20.0)	4 (6.7)
Weight decreased	12 (20.0)	1 (1.7)
Hypothyroidism	11 (18.3)	0
Vomiting	10 (16.7)	2 (3.3)
Decreased appetite	10 (16.7)	1 (1.7)
Hypoalbuminaemia	10 (16.7)	0
Hypokalaemia	9 (15.0)	1 (1.7)
Nausea	8 (13.3)	0
Occult blood positive	7 (11.7)	0
Haematuria	7 (11.7)	0
Back pain	7 (11.7)	0
Hyponatraemia	6 (10.0)	5 (8.3)
Bilirubin conjugated increased	6 (10.0)	3 (5.0)
Constipation	6 (10.0)	0

Data are shown in n (%).

### Efficacy

**Figure 4:**
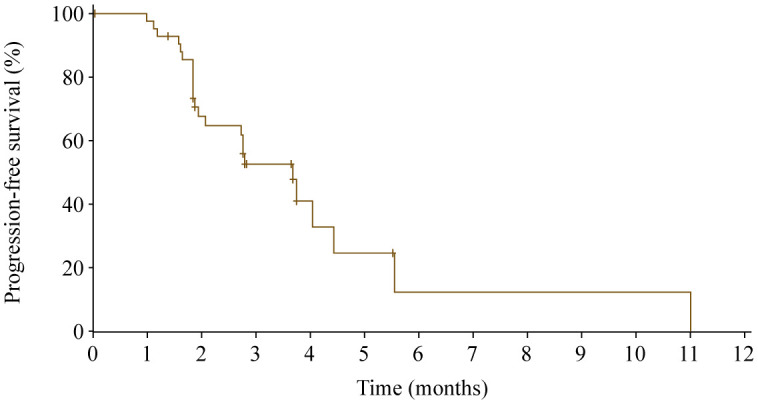
Progression-free survival.

At data cutoff, 17 patients were excluded from the efficacy set (nine patients with no post baseline tumor assessment, one patient with treatment duration lasting less than 2 weeks, and seven patients with both). One patient achieved partial response, and the ORR was 2.3% (95% CI 0.1%−12.3%). Sixteen patients had stable disease, and the DCR was 39.5% (95% CI 25.0%−55.6%). Twenty-four of 43 patients (55.8%) experienced death or disease progression, with a median PFS of 3.7 months (95% CI 2.1−4.4 months; **Figure 4**). The 6-month PFS rate was 12.3% (95% CI 1.0%−38.2%).

## Discussion

This study evaluated PK, safety, and antitumor activity of apatinib in patients with advanced gastric adenocarcinoma who had failed at least one line of previous chemotherapy. The dose was up-titrated from a starting dose of 500 mg once daily, up to 750 mg, and finally up to a maximum dose of 850 mg.

PK data demonstrated that C_max_ was achieved about 3.0 to 4.0 h after administration. After multiple dosing, only mild accumulation of apatinib was observed. These findings were consistent with the previous phase 1 study (13). Apatinib exposure (C_max_ and AUC_ss_) appeared to increase in a dose-dependent manner over the studied dose range, but the increase was less proportional than dose increase, which was consistent with the previous report (14). The plasma exposure showed high interpatient variability in patients with advanced gastric cancer, with the CV% both higher than 50% for C_max_ and AUC_ss_, and previous studies provided similar results (13, 14). The high interpatient variability in hepatic expression of CYP3A4/5, the major metabolizing enzyme for apatinib, might contribute to such high interpatient variability in PK profile (15, 16). In addition, the oral administration might also increase the interpatient variability of apatinib as reported in other TKIs (17).

Gastric resection results in functional and anatomical changes in the gastrointestinal tract, such as lack of acidic environment, reduced absorption area, and decreased gastric emptying rate, which might have an impact on drug absorption (14, 18, 19). In addition, a preclinical study demonstrated that in mice with gastrectomy, drug effectiveness was reduced because the expression levels of various CYPs in the liver were increased (20). This trial further evaluated the effect of gastric surgery on drug exposure in patients with advanced gastric cancer. In the present study, 45 (78.9%) of the 57 PK evaluable patients had undergone total or partial gastrectomy, which was comparable with the reports of previous phase 2 and 3 studies (2, 5). We observed an association between gastric resection history with apatinib exposure. After single (500 mg day 1) and multiple (500 mg day 14) dosing, geometric mean AUC was significantly lower in patients with total gastrectomy than patients with partial or no gastrectomy (*p* = 0.001 and 0.010, respectively). AUC values in patients with total gastrectomy only accounted for 48.9% and 55.2% of that in patients with no gastrectomy after single and multiple dosing. Notably, geometric mean AUC values were similar between patients with partial gastrectomy and those with no gastrectomy. Based on these data, we suggested that in the future clinical practice, the gastric resection status of patient should be taken into account in apatinib dosage selection. A population pharmacokinetic analysis of apatinib exhibited lower bioavailability and, consequently, decreased exposure in patients with advanced gastric cancer, all had undergone gastric surgery, than patients with other types of solid tumor (colorectal cancer, hepatic cancer, and breast cancer) (14). The results were consistent with our finding that advanced gastric cancer patients with gastrectomy exhibited a lower drug concentration.

We also investigated drug exposure in male and female patients. No apparent difference was observed, suggesting that it might not be necessary to determine the dosage according to gender.

Previous studies have reported that early presence of hypertension, hand-foot syndrome and proteinuria, the most commonly reported adverse events of apatinib, was correlated with antitumor activity of apatinib in metastatic gastric cancer (21), and treatment outcomes of TKIs were considered related to their exposure (17). Therefore, dose individualization based on drug exposure is necessary to improve clinical outcomes. Given that the PK profile analysis is patient unfriendly, our study firstly explored treatment optimization through a toxicity-based dose titration method. In the present study, 25 patients (41.7%) were up-titrated to 850 mg, and 26 patients (43.3%) failed to receive the highest dose due to adverse events. The 21 patients who completed all dose level plasma sampling had lower mean AUC_ss_ than the 57 PK evaluable patients at 500 mg day 1 (2980 h·ng/mL *vs* 4210 h·ng/mL), the 21 patients had lower mean AUC_ss_ than the 49 PK evaluable patients at 500 mg day 14 (4270 h·ng/mL *vs* 5480 h·ng/mL), and the 21 patients had lower mean AUC_ss_ than the 36 evaluable patients at 750 mg day 14 (5420 h·ng/mL *vs* 6560 h·ng/mL). Similar results for C_max_ were also observed. Adverse events grouped by maximum dose received showed that a higher percentage of patients not up-titrated to 850 mg experienced grade ≥3 adverse event and serious adverse events than those up-titrated to 850 mg, and more patients experienced dose delay or reduction and dose discontinuation or witdrawal in the 500 mg and 750 mg cohort than 850 mg cohort. These findings revealed that the lower exposure in those patients up-titrated to 850 mg might largely contributed to their high dose tolerance, and more importantly, demonstrated that a toxicity-based dose titration could effectively help to determine optimal dosage of apatinib for individual patients in clinical practice where monitoring plasma-drug concentration is mostly unavailable.

The safety profile was similar with that previously reported in the phase 2 and phase 3 studies (2, 5). Hypertension, hand-foot syndrome and proteinuria are the most common adverse events of antiangiogenic agents (22–24). In the present study, grade 3 or worse hypertension, hand-foot syndrome and proteinuria occurred in 11.7%, 8.3%, and 5.0% of patients, which were comparable with the previous reports (2, 5).

Although not a primary objective of this study, the antitumor activity of apatinib was investigated. The median PFS was 3.7 months (95% CI 2.1−4.4 months), and DCR was 39.5% (95% CI 25.0%−55.6%). These findings are consistent with results of the previous phase 2 and phase 3 study, with a median PFS of 2.6 to 3.7 months and a DCR of 31.8% to 51.1% in patients with advanced gastric cancer progressed on at least two lines of chemotherapy when given apatinib 850 mg once daily (2, 5).

## Conclusion

Data from the present study showed that in patients with advanced gastric adenocarcinoma, apatinib exposure (C_max_ and AUC_ss_) increased in a dose-dependent manner over the 500 mg to 850 mg dose range with a higher interpatient variability. Patients who were up-titrated to 850 mg had lower drug exposure than those who were not. Apatinib exposure was significantly lower in patients who underwent total gastrectomy compared with those who had partial or no gastrectomy. Our findings demonstrated that dose titration according to toxicity could reduce clinical risk and optimize individual apatinib therapy.

## Data availability statement

The datasets used and/or analyzed during the current study are available from the corresponding author on reasonable request. 

## Ethics statement

This study was reviewed and approved by Ethics Committee of Shanghai JiaoTong University Affiliated Sixth People’s Hospital; Ethics Committee of The First Hospital of Jilin University; Ethics Committee of Harbin Medical University Cancer Hospital; Ethics Committee of The First Affiliated Hospital of Anhui Medical University; Ethics Committee of Hunan Cancer Hospital, The Affiliated Cancer Hospital of Xiangya School of Medicine; Ethics Committee of The Affiliated Cancer Hospital of Zhengzhou University, Henan Cancer Hospital; Ethics Committee of Renmin Hospital of Wuhan University; Ethics Committee of Zhongshan Hospital Fudan University; Ethics Committee of Xinxiang Central Hospital; Ethics Committee of Changhai Hospital, Second Military Medical University; Ethics Committee of Xiangya Hospital Central South University; Ethics Committee of Shanghai East Hospital, Tongji University School of Medicine. The patients/participants provided their written informed consent to participate in this study.

## Author contributions

YG and YY conceived and designed the study. YW, CW, YQZ, JH, NY, JW, MP, TL, GZ, XZ, SZ, YG, and YY enrolled patients and collected the data. YFZ analyzed and interpreted the data. Manuscript was drafted by YW and CW, and was reviewed and/or revised by all authors. All authors contributed to the article and approved the submitted version.

## Acknowledgments

We are grateful to all patients and their families and all members of the collaborative group in this trial. Medical writing support was provided by Yanwen Wang (Hengrui Pharmaceuticals) according to Good Publication Practice Guidelines.

## Conflict of interest

XZ has served on the speaker's bureau for Lilly, Bayer, and Hengrui Pharmaceuticals. The authors declare that this study received funding from Jiangsu Hengrui Pharmaceuticals. The funder had the following involvement in the study: study design, data collection, data analysis, interpretation of data, and writing of this article.

The remaining authors declare that the research was conducted in the absence of any commercial or financial relationships that could be construed as a potential conflict of interest.

## Publisher’s note

All claims expressed in this article are solely those of the authors and do not necessarily represent those of their affiliated organizations, or those of the publisher, the editors and the reviewers. Any product that may be evaluated in this article, or claim that may be made by its manufacturer, is not guaranteed or endorsed by the publisher.

## References

[1] Tian S Quan H Xie C Guo H Lü F Xu Y. YN968D1 is a novel and selective inhibitor of vascular endothelial growth factor receptor-2 tyrosine kinase with potent activity *in vitro* and *in vivo* . Cancer Sci (2011) 102(7):1374–80. doi: 10.1111/j.1349-7006.2011.01939.x PMC11158267 21443688

[2] Li J Qin S Xu J Xiong J Wu C Bai Y. Randomized, double-blind, placebo-controlled phase III trial of apatinib in patients with chemotherapy-refractory advanced or metastatic adenocarcinoma of the stomach or gastroesophageal junction. J Clin Oncol (2016) 34(13):1448–54. doi: 10.1200/JCO.2015.63.5995 26884585

[3] Cheng AL Kang YK Chen Z Tsao C-J Qin S Kim JS. Efficacy and safety of sorafenib in patients in the Asia-pacific region with advanced hepatocellular carcinoma: a phase III randomised, double-blind, placebo-controlled trial. Lancet Oncol (2009) 10(1):25–34. doi: 10.1016/S1470-2045(08)70285-7 19095497

[4] Raymond E Dahan L Raoul J-L Bang Y-J Borbath I Lombard-Bohas C. Sunitinib malate for the treatment of pancreatic neuroendocrine tumors. N Engl J Med (2011) 364(6):501–13. doi: 10.1056/NEJMoa1003825 21306237

[5] Li J Qin S Xu J Guo W Xiong J Bai Y. Apatinib for chemotherapy-refractory advanced metastatic gastric cancer: results from a randomized, placebo-controlled, parallel-arm, phase II trial. J Clin Oncol (2013) 31(26):3219–25. doi: 10.1200/JCO.2013.48.8585 23918952

[6] Zhang Y Han C Li J Zhang L Wang L Ye S. Efficacy and safety for apatinib treatment in advanced gastric cancer: a real world study. Sci Rep (2017) 7(1):13208. doi: 10.1038/s41598-017-13192-8 29038432 PMC5643341

[7] Lin H Han D Fu G Liu C Wang L Han S. Concurrent apatinib and docetaxel vs apatinib monotherapy as third-or subsequent-line therapy for advanced gastric adenocarcinoma: A retrospective study. Onco.Tar. Ther (2019) 12:1681–9. doi: 10.2147/OTT.S193801 PMC6400117 30881023

[8] Ruan H Dong J Zhou X Xiong J Wang H Zhong X. Multicenter phase II study of apatinib treatment for metastatic gastric cancer after failure of second-line chemotherapy. Oncotarget (2017) 8(61):104552–9. doi: 10.18632/oncotarget.21053 PMC5732826 29262660

[9] Du Y Cao Q Jiang C Liang H Ning Z Ji C. Effectiveness and safety of low-dose apatinib in advanced gastric cancer: A real-world study. Cancer Med (2020) 9:5008–14. doi: 10.1002/cam4.3105 PMC7367613 32441892

[10] Hu X Cao J Hu W Wu C Pan Y Cai L. Multicenter phase II study of apatinib in non-triple-negative metastatic breast cancer. BMC Cancer (2014) 14(1):820. doi: 10.1186/1471-2407-14-820 25376790 PMC4237755

[11] Kong Y Sun L Hou Z Zhang Y Chen P Cui Y. Apatinib is effective for treatment of advanced hepatocellular carcinoma. Oncotarget (2017) 8(62):105596–605. doi: 10.18632/oncotarget.22337 PMC5739662 29285275

[12] Wu F Zhang S Xiong A Gao G Li W Cai W. A phase II clinical trial of apatinib in pretreated advanced non-squamous non-small-cell lung cancer. Clin Lung Cancer (2018) 19(6):e831–42. doi: 10.1016/j.cllc.2018.06.002 30026059

[13] Li J Zhao X Chen L Guo H Lv F Jia K. Safety and pharmacokinetics of novel selective vascular endothelial growth factor receptor-2 inhibitor YN968D1 in patients with advanced malignancies. BMC Cancer (2010) 10(1):529. doi: 10.1186/1471-2407-10-529 20923544 PMC2984425

[14] Yu M Gao Z Dai X Gong H Zhang L Chen X. Population pharmacokinetic and covariate analysis of apatinib, an oral tyrosine kinase inhibitor, in healthy volunteers and patients with solid tumors. Clin Pharmacokinet (2017) 56(1):65–76. doi: 10.1007/s40262-016-0427-y 27379402

[15] Westlind-Johnsson A Malmebo S Johansson A Otter C Andersson TB Johansson I. Comparative analysis of CYP3A expression in human liver suggests only a minor role for CYP3A5 in drug metabolism. Drug Metab Dispos (2003) 31(6):755–61. doi: 10.1124/dmd.31.6.755 12756208

[16] Ding J Chen X Gao Z Dai X Li L Xie C. Metabolism and pharmacokinetics of novel selective vascular endothelial growth factor receptor-2 inhibitor apatinib in humans. Drug Metab Dispos (2013) 41(6):1195–210. doi: 10.1124/dmd.112.050310 23509226

[17] de Wit D Guchelaar H-J den Hartigh J Gelderblom H van Erp NP. Individualized dosing of tyrosine kinase inhibitors: Are we there yet? Drug Discovery Today (2015) 20(1):18–36. doi: 10.1016/j.drudis.2014.09.007 25245169

[18] Brocks DR Ben-Eltriki M Gabr RQ Padwal RS. The effects of gastric bypass surgery on drug absorption and pharmacokinetics. Expert Opin Drug Metab Toxicol (2012) 8(12):1505–19. doi: 10.1517/17425255.2012.722757 22998066

[19] Titus R Kastenmeier A Otterson MF. Consequences of gastrointestinal surgery on drug absorption. Nutr Clin Pract (2013) 28(4):429–36. doi: 10.1177/0884533613490740 23835364

[20] Ishii M Toda T Ikarashi N Kusunoki Y Kon R Ochiai W. Total gastrectomy may result in reduced drug effectiveness due to an increase in the expression of the drug-metabolizing enzyme cytochrome P450, in the liver. Eur J Pharma. Sci (2014) 51:180–8. doi: 10.1016/j.ejps.2013.09.017 24095864

[21] Liu X Qin S Wang Z Xu J Xiong J Bai Y. Early presence of anti-angiogenesis-related adverse events as a potential biomarker of antitumor efficacy in metastatic gastric cancer patients treated with apatinib: A cohort study. J Hematol Oncol (2017) 10(1):153. doi: 10.1186/s13045-017-0521-0 28870253 PMC5584332

[22] Ohtsu A Shah MA Van Cutsem E Rha SY Sawaki A Park SR. Bevacizumab in combination with chemotherapy as first-line therapy in advanced gastric cancer: a randomized, double-blind, placebo-controlled phase III study. J Clin Oncol (2011) 29(30):3968–76. doi: 10.1200/JCO.2011.36.2236 21844504

[23] Liu L Wu N Li J. Novel targeted agents for gastric cancer. J Hematol Oncol (2012) 5(1):31. doi: 10.1186/1756-8722-5-31 22709792 PMC3411478

[24] Fuchs CS Tomasek J Yong CJ Dumitru F Passalacqua R Goswami C. Ramucirumab monotherapy for previously treated advanced gastric or gastro-oesophageal junction adenocarcinoma (REGARD): An international, randomised, multicentre, placebo-controlled, phase 3 trial. Lancet (2014) 383(9911):31–9. doi: 10.1016/S0140-6736(13)61719-5 24094768

