# Supplementary material for: Multiple-dose up-titration study to evaluate the pharmacokinetics, safety and antitumor activity of apatinib in advanced gastric adenocarcinoma
Source: Front Oncol. 2022 Oct 18;12:876899. doi: 10.3389/fonc.2022.876899 (PMC9623328; doi:10.3389/fonc.2022.876899)
Supplement: Supplementary file 1 [file DataSheet_1.docx]

**Supplementary Material**

**Title:** Multiple-dose Up-titration Study to Evaluate the Pharmacokinetics, Safety and Antitumor Activity of Apatinib in Advanced Gastric Adenocarcinoma

**Content**

[Table 1 Patient disposition at the time of data cutoff 2](#_Toc111032443)

[Table 2 Multiple comparison of pharmacokinetics in patients with different extent of gastrectomy at 500 mg day 1 3](#_Toc111032444)

[Table 3 Multiple comparison of pharmacokinetics in patients with different extent of gastrectomy at 500 mg day 14 4](#_Toc111032445)

[Table 4 Gender difference in pharmacokinetics in patients with different extent of gastrectomy at 500 mg days 1 and 14 5](#_Toc111032446)

[Table 5 Patient distribution according to the maximum dose received 6](#_Toc111032447)

[Table 6 Apatinib exposure 7](#_Toc111032448)

[Table 7 Adverse events leading to dose up-titration halt 8](#_Toc111032449)

[Table 8 Adverse events leading to treatment discontinuation 9](#_Toc111032450)

# Table 1 Patient disposition at the time of data cutoff

| **Disposition** | **Total patients**  **(n = 60)** |
| --- | --- |
| Radiographic disease progression | 20 |
| Adverse events | 15 |
| Others^a^ | 14 |
| Withdrawal of consent | 7 |
| Death | 3 |
| Clinical progression | 1 |

^a^ 12 patients completed study treatment, one patient delayed treatment for 14 days, and one patient received other anticancer treatment.

# Table 2 Multiple comparison of pharmacokinetics in patients with different extent of gastrectomy at 500 mg day 1

| **Parameters** | **Group A** | **Group B** | **Mean difference**^a^ | **95% CI** | ***p* value** |
| --- | --- | --- | --- | --- | --- |
| C_max_ | Total | Partial | -0.4557 | -0.8229 to -0.0886 | 0.016 |
|  |  | No | -0.5280 | -0.9782 to 0.0779 | 0.022 |
|  | Partial | Total | 0.4557 | 0.0886 to 0.8229 | 0.016 |
|  |  | No | -0.0723 | -0.4843 to 0.3397 | 0.726 |
|  | No | Total | 0.5280 | 0.0779 to 0.9782 | 0.022 |
|  |  | Partial | 0.0723 | -0.3397 to 0.4843 | 0.726 |
| AUC_24_ | Total | Partial | -0.5822 | -0.9239 to -0.2405 | 0.001 |
|  |  | No | -0.7148 | -1.1338 to -0.2958 | 0.001 |
|  | Partial | Total | 0.5822 | 0.2405 to 0.9239 | 0.001 |
|  |  | No | -0.1326 | -0.5160 to 0.2509 | 0.491 |
|  | No | Total | 0.7148 | 0.2958 to 1.1338 | 0.001 |
|  |  | Partial | 0.1326 | -0.2509 to 0.5160 | 0.491 |

^a^ Mean difference represents group A *vs* group B. Abbreviations: Total, total gastrectomy; Partial, partial gastrectomy; No, no gastrectomy; CI, confidence interval

# Table 3 Multiple comparison of pharmacokinetics in patients with different extent of gastrectomy at 500 mg day 14

| **Parameters** | **Group A** | **Group B** | **Mean difference**^a^ | **95% CI** | ***p* value** |
| --- | --- | --- | --- | --- | --- |
| C_max_ | Total | Partial | -0.3443 | -0.7051 to 0.0165 | 0.061 |
|  |  | No | -0.5401 | -1.0849 to 0.0046 | 0.052 |
|  | Partial | Total | 0.3443 | -0.0165 to 0.7051 | 0.061 |
|  |  | No | -0.1958 | -0.7031 to 0.3115 | 0.441 |
|  | No | Total | 0.5401 | -0.0046 to 1.0849 | 0.052 |
|  |  | Partial | 0.1958 | -0.3115 to 0.7031 | 0.441 |
| AUC_ss_ | Total | Partial | -0.5338 | -0.8896 to -0.1779 | 0.004 |
|  |  | No | -0.5919 | -1.1291 to -0.0547 | 0.032 |
|  | Partial | Total | 0.5338 | 0.1779 to 0.8896 | 0.004 |
|  |  | No | -0.0581 | -0.5585 to 0.4422 | 0.816 |
|  | No | Total | 0.5919 | 0.0547 to 1.1291 | 0.032 |
|  |  | Partial | 0.0581 | -0.4422 to 0.5585 | 0.816 |

^a^ Mean difference represents group A *vs* group B. Abbreviations: Total, total gastrectomy; Partial, partial gastrectomy; No, no gastrectomy; CI confidence interval.

# Table 4 Gender difference in pharmacokinetics in patients with different extent of gastrectomy at 500 mg days 1 and 14

| **Dose** | **Extent of gastrectomy** | **Parameters** | **No. of male** | **No. of female** | **Geometric mean** | | **Geometric mean ratio**  **(male/female)** | **90% CI** | ***p* value** |
| --- | --- | --- | --- | --- | --- | --- | --- | --- | --- |
|  |  |  |  |  | **Male** | **Female** |  |  |  |
| 500 mg day 1 | Total | AUC_24_ (h·ng/mL) | 14 | 3 | 2430 | 1690 | 1.44 | 0.78 to 2.64 | 0.311 |
|  |  | C_max_ (ng/mL) |  |  | 247 | 175 | 1.42 | 0.71 to 2.83 | 0.393 |
|  | Partial | AUC_24_ (h·ng/mL) | 20 | 8 | 4380 | 3420 | 1.28 | 0.88 to 1.87 | 0.272 |
|  |  | C_max_ (ng/mL) |  |  | 380 | 336 | 1.13 | 0.76 to 1.68 | 0.592 |
|  | No | AUC_24_ (h·ng/mL) | 8 | 4 | 4510 | 4990 | 0.90 | 0.45 to 1.84 | 0.801 |
|  |  | C_max_ (ng/mL) |  |  | 350 | 500 | 0.70 | 0.33 to 1.50 | 0.416 |
| 500 mg day 14 | Total | AUC_ss_ (h·ng/mL) | 13 | 2 | 3300 | 2310 | 1.42 | 0.78 to 2.61 | 0.319 |
|  |  | C_max_ (ng/mL) |  |  | 320 | 301 | 1.06 | 0.53 to 2.16 | 0.878 |
|  | Partial | AUC_ss_ (h·ng/mL) | 20 | 8 | 5100 | 6080 | 0.84 | 0.54 to 1.30 | 0.500 |
|  |  | C_max_ (ng/mL) |  |  | 419 | 532 | 0.79 | 0.52 to 1.19 | 0.333 |
|  | No | AUC_ss_ (h·ng/mL) | 3 | 3 | 7200 | 4490 | 1.61 | 0.70 to 3.69 | 0.292 |
|  |  | C_max_ (ng/mL) |  |  | 801 | 371 | 2.16 | 0.94 to 4.96 | 0.120 |

Abbreviations: Total, total gastrectomy; Partial, partial gastrectomy; No, no gastrectomy; CI, confidence interval.

# Table 5 Patient distribution according to the maximum dose received

| **Dose cohort** | **n (%)** | **Distribution** |
| --- | --- | --- |
| 500 mg | 17 (28.3) | Adverse events (n = 13)  Disease progression (n = 1)  Patient decision (n = 2)  Death (n = 1) |
| 750 mg | 18 (30.0) | Adverse events (n = 13)  Disease progression (n = 1)  Patient decision (n = 1)  Patient request to discontinue study treatment (n = 1)  Protocol violation (n = 2) |
| 850 mg | 25 (41.7) | — |

# **Table 6** Apatinib exposure

|  | **500 mg cohort**  **(n = 17)** | **750 mg cohort**  **(n = 18)** | **850 mg cohort**  **(n = 25)** |
| --- | --- | --- | --- |
| Treatment duration (days) | | | |
| Mean ± SD | 32 ± 31 | 79 ± 47 | 94 ± 64 |
| Range | 3‒112 | 21‒174 | 30‒335 |
| Dose received (mg) | | | |
| Mean ± SD | 469 ± 60 | 575 ± 78 | 720 ± 80 |
| Range | 269‒500 | 442‒717 | 486‒810 |

Abbreviations: SD, standard deviation.

# Table 7 Adverse events leading to dose up-titration halt

| **Adverse events** | **Total patients**  **(n = 60)** |
| --- | --- |
| Neutrophil count decreased | 3 (5.0) |
| Hand-foot syndrome | 3 (5.0) |
| Asthenia | 3 (5.0) |
| Hypertension | 2 (3.3) |
| Death | 2 (3.3) |
| White blood cell count decreased | 2 (3.3) |
| Platelet count decreased | 2 (3.3) |
| Gamma-glutamyltransferase increased | 2 (3.3) |
| Electrocardiogram QT prolonged | 1 (1.7) |
| Blood thyroid stimulating hormone increased | 1 (1.7) |
| Intestinal obstruction | 1 (1.7) |
| Abdominal pain | 1 (1.7) |
| Abdominal distension | 1 (1.7) |
| Upper gastrointestinal haemorrhage | 1 (1.7) |
| Gastrointestinal haemorrhage | 1 (1.7) |
| Gastrooesophageal reflux disease | 1 (1.7) |
| Dyspepsia | 1 (1.7) |
| Cerebral haemorrhage | 1 (1.7) |
| Neurotoxicity | 1 (1.7) |
| Cerebral infarction | 1 (1.7) |
| Deep vein thrombosis | 1 (1.7) |
| Back pain | 1 (1.7) |
| Arthralgia | 1 (1.7) |
| Bone marrow failure | 1 (1.7) |
| Anaemia | 1 (1.7) |
| Hypokalaemia | 1 (1.7) |
| Hepatic function abnormal | 1 (1.7) |

Data are shown in n (%).

# Table 8 Adverse events leading to treatment discontinuation

|  | **Total patients**  **(n = 60)** |
| --- | --- |
| Subileus | 1 (1.7) |
| Intestinal obstruction | 1 (1.7) |
| Nausea | 1 (1.7) |
| Abdominal discomfort | 1 (1.7) |
| Abdominal distension | 1 (1.7) |
| Vomiting | 1 (1.7) |
| Upper gastrointestinal haemorrhage | 1 (1.7) |
| Gastric perforation | 1 (1.7) |
| Gastrooesophageal reflux disease | 1 (1.7) |
| Dyspepsia | 1 (1.7) |
| Gamma-glutamyltransferase increased | 1 (1.7) |
| Alanine aminotransferase increased | 1 (1.7) |
| Aspartate aminotransferase increased | 1 (1.7) |
| Blood alkaline phosphatase increased | 1 (1.7) |
| Cerebral infarction | 1 (1.7) |
| Decreased appetite | 1 (1.7) |
| Hepatic function abnormal | 1 (1.7) |
| Hypothyroidism | 1 (1.7) |
| Deep vein thrombosis | 1 (1.7) |

Data are shown in n (%).
